# Predictors and Correlates of Fatigue in Sickle Cell Disease Patients

**Published:** 2018-01-01

**Authors:** Mehrnaz Ahmadi, Saeed Poormansouri, Samira Beiranvand, Ladan Sedighie

**Affiliations:** 1 Department of Nursing, Student Research Committee, School of Nursing and Midwifery, Ahvaz Jundishapur University of Medical Sciences, Ahvaz, Iran; 2 Treatment Deputy, Ahvaz Jundishapur University of Medical Sciences, Ahvaz, Iran; 3 Department of Nursing, Student Research Committee, School of Nursing and Midwifery, Ahvaz Jundishapur University of Medical Sciences, Ahvaz, Iran; 4 Department of Nursing, Student Research Committee, School of Nursing and Midwifery, Shahid Beheshti University of Medical Sciences, Tehran, Iran

**Keywords:** Anxiety, Depression, Stress, Fatigue, Sickle cell disease

## Abstract

**Background: **Although fatigue is the most important symptom of Sickle Cell Disease, the extent of it is unknown, and causal mechanisms are not well understood. This article explores biopsychosocial characteristics that can potentially contribute to fatigue in SCD.

**Materials and Methods: **This cross-sectional, correlational study included 97 SCD patients who aged over 16 years and had records in Thalassemia Ward and Clinic of Shafa Hospital affiliated to Ahvaz University of Medical Sciences, Ahvaz, Iran. Data were collected from a self- reported demographic questionnaire, measuring depression, anxiety stress scale (DASS-21) and fatigue severity scale (FSS). Data analysis was done by descriptive statistics, independent sample t-tests, Pearson's correlation coefficient, one-way ANOVA and multiple stepwise regression.

**Results: **More than 50% of study participants were mostly single women. A majority of patients had a diagnosis of HgbSS disease. Levels of depression, anxiety and stress were severe in more than half of the participants. About 65% of SCD patients reported signs of fatigue. Moreover, fatigue, depression, anxiety and stress had a high intercorrelation. Depression, blood transfusion, renal diseases and work status were predictors of fatigue according to the models used in this survey.

**Conclusion: **The results of the study indicated that SCD patients who had depression, blood transfusions, SCD-related renal complications, students and working people experienced more fatigue. So, if fatigue is present, it is important to recognize the existence of these conditions or vice versa. Routine assessment and improved management of fatigue, effective interventions to reduce fatigue, are highly recommended for patients with SCD.

## Introduction

 Sickle cell disease (SCD) is the most common genetic disease characterized by recurrent painful vaso- occlusive crises^1^. Although the most frequent symptom associated with SCD is pain and historically and justifiably investigators have focused on improving sickle cell pain, fatigue is an increasingly important symptom of this disease in the literature. The extent of fatigue in SCD is still unknown, and causal mechanisms are not well understood^2^^,^^3^. On the other hand, several symptoms of SCD such as the chronic hemolytic anemia, inflammation, and pain suggest that individuals with SCD are at risk for experiencing acute and chronic fatigue, so that researchers of cancer-related fatigue have demonstrated that a systematic approach to the investigation of this symptom is necessary for understanding this phenomenon in SCD^4^. In addition, recent studies have shown that patients with SCD suffer from numerous psychosocial problems such as depression^5^^,^^6^, anxiety and stress^5^^, ^^7^^-^^12^ leading to fatigue^3^^- ^^4^^, ^^13^ and poor quality of life^14^^-^^16^. Moreover, several individual and disease-related factors including age, sex, ethnicity, socioeconomic status, treatments, disease severity, time with disease, medications, long-term complications, and cumulative organ damage may contribute to fatigue ^3^^,^^4^^, ^^10^^- ^^12^. There are little knowledge about the exact relationship between fatigue and these factors in SCD, and so these relationships need to be better understood. Meanwhile, SCD is more prevalent in some parts of Iran including Khuzestan province (particularly Ahvaz, southwestern province of Khuzestan, Abadan, etc) and immediate medical attention is required^17^. Hence, this paper attempts 1) to describe fatigue; 2) to examine the relationships between fatigue and depression, anxiety, stress, individual and disease characteristics; and 3) to find whether depression, anxiety, stress, demographic factors and other variables in this study could predict the fatigue in patients with SCD.

## Materials and Methods

**Participants**

This cross-sectional, descriptive, correlational study was approved by the Institution's Review Board (Ethic Code ETH-739). The study group included 168 patients who were diagnosed with SCD based on their records in Thalassemia Ward and Clinic of Shafa Hospital affiliated to Ahvaz University of Medical Sciences between 2011 to 2013. Inclusion criteria were: ages over 16 years and SCD diagnosis. Data were collected through census method of the entire population. Among 128 patients referred to clinic for daily visit within six months from February 2013 to July 2013, 110 entered the study; of whom 13 either did not return the form or failed to complete it in an appropriate way, so finally the study participants consisted of 97 adults with SCD aged more than 16 years (Table 1). Written informed consent was obtained from the participants prior to their participation in the study. 

**Table 1:** Demographic features of participants in the study

**Demographic profile**		**frequency**	**%**
sex	female	63	64.9
	male	34	35.1
Age	16-24	53	54.6
	≥25	44	45.4
Marital status	Singles	66	68
	Married	30	31
	Divorced	1	1
Ethnicity	Arab	92	94.8
	Fars	5	5.2
Educational level	<High school Diploma	50	51.6
	High school Diploma	36	37.1
	college	11	11.3
Work status	Not employed	55	56.7
	Employed	22	22.6
	Student	20	20.6
Income per month (Rial)	> 5000000	82	84.6
	5000000-10000000	12	12.4
	<10000000	3	3.1
Sickle cell disease genotype	HbSS	63	64.9
	Sickle Beta Thalassemia	34	35.1

**Instruments**

**Demographic questionnaire**

The demographic information questionnaire included items such as age, sex, ethnicity, marital status, level of education, work status, income, sickle cell disease genotype, blood transfusion, hydroxyl urea, Splenic, heart, renal, bone and joint disease. 

**Depression, Anxiety and Stress**

The Persian version of DASS-21, a validated and structured questionnaire, was used to assess depression, anxiety and stress level^18^^.^ DASS-21 consisted of 21 questions divided into 3 subscales of 7 items: depression, anxiety, and stress. Participants rated the extent to which they experienced each symptom on a 4-point scale, ranging from 0 (does not apply to me at all) to 3 (applies to me most or all of the time). Depression (0 to 4, 5 to 6, 7 to 10, and 11-21), anxiety (0 to 3, 4 to 5, 6 to 7, and 8-21), and stress (0 to 7, 8 to 9, 10 to 12, and 13-21) scores were divided into normal, mild, moderate, and severe, respectively. The questionnaires were conducted or supervised by a trained registered nurse or researchers, and the identity of the participants was undisclosed.

**Fatigue**

The Fatigue Severity Scale (FSS) is a 9-item self-report scale from 0 (lack of fatigue) to 9 (severe fatigue) that assesses the severity of fatigue and has evidence of being a reliable and valid measure^19^. A total fatigue score was obtained by summing the 9 item scores. The higher and lower scores from 36 indicated the patients with and without signs of fatigue, respectively. This measure assessed all dimensions of fatigue (physical, mental, emotional, behavioral and social). 

**Procedure**

Recruitment took place at the Adult Hematology Clinics and Thalassemia Ward. The details of the study were explained to all potential participants and their families. Written informed consent was obtained from adult participants. During a routine clinic visit or hospitalization, participants were asked to complete the questionnaire.

**Data analysis**

Data analysis was conducted using SPSS statistical software version 19. Descriptive statistics (frequency, mean and standard deviation) were calculated. Kolmogorov-Smirnov was used to examine the normal distribution of variables. Independent-samples t-test and one-way Anova were used to compare the mean scores of fatigue, depression, anxiety and stress in terms of dichotomous and clinical variables. Moreover, independent-samples t-test was used to compare fatigue status in SCD patients with and without sign of depression, anxiety and stress. Pearson correlation coefficients were calculated to examine the relationship between fatigue and depression, anxiety and stress. Then, multiple stepwise regression models were fitted to test the independent association of depression, anxiety, stress and demographics and clinical variables with fatigue as dependent variable.

## Results

 Ninety-seven SCD patients, aged over 16 years, participated in the study. Sample characteristics are reported in Table 1. More than 50% of study participants were mostly single women. A majority of study participants had a diagnosis of HgbSS disease. 

Descriptive results for the behavioral variables (depression, anxiety, stress and fatigue) are illustrated in Table 2. As shown in Table 3, self-reported levels of depression, anxiety, and stress were severe in more than half of the participants. About 65% of SCD patients reported signs of fatigue. Results from independent- samples t-test showed those who had symptomatic depression and stress experienced more fatigue. The same relationship was not observed regarding anxiety (Table 3). Fatigue, depression, anxiety and stress had a high correlation based on Pearson's correlation coefficient (Table 4). The results of independent-samples t-test and one-way Anova analysis between the fatigue, depression, anxiety, stress and study variables were displayed through Table 5. Finally, prior results provided situation for regression analysis. In this study, we used the method of stepwise multiple regression for data analysis in order to find whether depression, anxiety, stress, demographic factors and other variables could predict the fatigue in patients with SCD (Table 6).

Our finding in the first model demonstrated that depression plays the most significant role in predicting fatigue in these patients. The correlation coefficient between depression and fatigue was reported 0.43, predicting 18% fatigue in these patients. In the second model, after depression, blood transfusion entered the equation, so the correlation coefficient between these two variables with fatigue was reported 0.53, predicting 28% changes in SCD fatigue.

**Table 2:** Depression, anxiety, stress and fatigue Status in SCD Patients

**Variable**	**Mean ± SD**	**Level**					**Asymptomatic** **No (%)**	**Symptomatic** **No (%)**
		**Normal** **No (%)**	**Mild** **No (%)**	**Moderate** **No (%)**	**Sever** **No (%)**			
Depression	8.67±5.53	32 (33)	9 (9.3)	10 (10.3)	46 (47.4)		32 (33)	65 (67)
Anxiety	8.59±4.6	8 (8.2)	16 (16.5)	19 (19.6)	54 (55.6)		8 (8.9)	89 (91.8)
Stress	11. 81±4.9	14 (14.4)	10 (10.3)	28 (28.9)	45 (46.4)		14 (14.4)	83 (85.6)
		Fatigue				NO (%)		
Fatigue	38.56±14.88	No signs of fatigue <36				34 (35.1)		
		With signs of fatigue >36				63 (64.9)		

**Table 3:** A Comparison of fatigue status in SCD patients with and without sign of depression, anxiety and stress

**Variable**		**Fatigue (M±SD)**	**P**
Depression	Asymptomatic	32.43±13.5	0.004
	Symptomatic	41.58±14.6	
Anxiety	Asymptomatic	40± 17.7	0.1
	Symptomatic	39. 8±13	
Stress	Asymptomatic	32±13.9	0.01
	Symptomatic	41.1±12.8	

**Table 4:** A Correlation between fatigue and depression, anxiety and stress

	**Depression**	**Anxiety**	**stress**
	r (pvalue)	r (pvalue)	r (pvalue)
Fatigue	0.43 (<0.001)	0.26 (0.009)	0.34 (0.001)

**Table 5:** A Comparison of depression, anxiety, stress and fatigue in the samples based on demographic variables and disease comorbidities

**Variables**		**Fatigue** **M±SD**	**Depression** **M±SD**	**Anxiety** **M±SD**	**Stress** **M±SD**
Age	16-24	38.3±12.8	8.3±5.2	8.1±4.3	11.6±4.8
	≤25	38.7±17.1	9±5.8	9±4.8	12±5
	P value	0.9	0.4	0.3	0.6
Sex	male	38.7±14	8.4±5.6	7.8±4.1	10.7±4.9
	female	38.4±15.4	8.8±5.5	8.9±4.8	12.4±4.8
	P value	0.9	0.7	0.2	0.1
Marital status	Singles	39±14	8.9±5.4	8.8±4.6	12±4.6
	Married	37.5±16.8	8±5.7	8±4.6	11.2±5.4
	P value	0.6	0.4	0.3	0.4
Work status	Un employed	28.2±14.2	9.6±5.5	9.7±4.7	12.9±5.2
	Employed	38.3±13.5	8±4.6	7.9±3.8	10.4±2.8
	student	47.5±13.7	8.5±6	7.7±3.5	12.1±3.3
	P value	0.04	0.4	0.1	0.08
Educational level	≤High school Diploma	39.1±14.6	8.7±5.5	8.5±4.6	11.8±5
	College	33.7±16.6	8.3±5.5	8.6±4.1	11.2±4.2
	P value	0.2	0.8	0.9	0.7
Sickle cell disease genotype	HbSS	38±15.4	8±5.1	8.1±4.6	11.4±4.9
	Sickle Beta Thalassemia	39.5±13.9	9.8±6	9.4±4.4	12.5±4.7
	P value	0.6	0.1	0.1	0.2
Blood transfusion	yes	41±14.7	9.3±5.5	8.7±4.5	12.3±4.6
	no	28±11	5.8±4.4	8±4.9	9.6±5.5
	P value	0.001	0.007	0.5	0.03
Hydroxyl urea	yes	38.4±14.4	8.1±5.3	8.3±4.3	11.3±4
	no	38.6±15.3	9.1±5.7	8.8±4.8	12.2±5.5
	P value	0.9	0.3	0.6	0.3
Splenic disease	yes	37.3±14.7	8.3±5.1	7.8±4.6	10.6±5.1
	no	39.8±15	8.9±5.8	9.3±4.4	12.9±4.4
	P value	0.4	0.6	0.1	0.02
Bone and joint disease	yes	39.4±11	8.8±4.7	9.1±4.4	11.9±4
	no	38.4±15.5	8.6±5.6	8.5±4.6	11.7±5
	P value	0.8	0.8	0.6	0.9
Heart disease	yes	37.5±14.9	8.3±5.4	7.8±4.2	11.1±4.8
	no	44.6±13.7	10.7±5.9	12.9±4.3	15.8±2.8
	P value	0.09	0.1	<0.001	0.001
Renal disease	yes	37.1±14.6	8.4±5.4	8.1±4.5	11.6±5
	no	45±14.5	9.8±5.9	10.7±4.2	12.7±4.5
	P value	0.04	0.3	0.03	0.4

**Table 6:** Results of regression analysis with stepwise model

**Predictor**	**R**	**R** ^2^	**Adj.** ***R*** ^2^	**F**	**df1**	**df2**	**B**	**SE**	**β**	**t**	**p**
Model 1	0.43	0.185	0.177	21.59	1	95	-	-	-	-	<0.001
Constant	-	-	-	-	-	-	30.279	2.398	-	12.62	<0.001
Dep	-	-	-	-	-	-	1.056	0.227	0.43	4.646	<0.001
*Model 2*	0.533	0.284	0.268	18.60	2	94	-	-	-	-	<0.001
Constant	-	-	-	-	-	-	45.49	4.80	-	9.47	<0.001
Depression	-	-	-	-	-	-	0.824	0.224	0.336	3.68	<0.001
Blood transfusion	-	-	-	-	-	-	-10.96	3.05	-0.328	-3.59	0.001
*Model 3*	0.575	0.330	0.309	15.29	3	93	-	-	-	-	<0.001
Constant	-	-	-	-	-	-	44.39	4.68	-	9.47	<0.001
Depression	-	-	-	-	-	-	0.748	0.219	3.40	-3.56	0.001
Blood transfusion	-	-	-	-	-	-	-10.59	2.96	-0.317	-3.56	0.001
Renal disease	-	-	-	-	-	-	7.66	3	0.219	2.55	0.012
Model 4	0.601	0.361	0.333	12.99	4	92	-	-	-	-	<0.001
Constant	-	-	-	-	-	-	37.70	5.59	-	6.73	<0.001
Depression	-	-	-	-	-	-	0.821	0.218	0.335	3.76	<0.001
Blood transfusion	-	-	-	-	-	-	-9.54	2.95	-0.285	-3.22	0.002
Renal disease	-	-	-	-	-	-	7.14	2.96	0.205	2.41	0.018
Employment	-	-	-	-	-	-	2.96	1.41	0.179	2.09	0.039

Actually, blood transfusion variable could increase the predictive power about 10 %. In the third model, renal disease was added as the third predictor variable and could increase the predictive power at approximately 5%. In other words, these three variables together predict 33 % of the variations in fatigue. The fourth variable imported into the regression analysis equation was working status in patients which could enhance fatigue prediction power about 36% (an increase about 3 %). In total, the correlation coefficient between all above- mentioned variables and fatigue was 0.6. Regarding the arrangement of entering the variables in fourth model and the standardized (beta) regression coefficient of the regression equation, depression with standard beta 0.335 had the highest share in predicting changes in fatigue of SCD patients. Then, blood transfusion with standard beta -0.285 played negative significant role in patients with fatigue state. In subsequent steps, renal disease with beta 0.205 and work status with beta 0.179, in terms of prediction of fatigue changes in SCD patients, were placed in the next ranks, respectively.

Finally, this model indicated that the rise in depression is associated with increased level of fatigue by as much as 0.82. Patients who did not receive blood transfusion were 9.5 times more likely to experience fatigue compared to those receiving blood transfusion. Those who suffered from SCD-related renal complications were 1.7 times more likely to experience fatigue. Lastly, students and employed people were 2.9 times more likely to experience fatigue compared to employed and unemployed people, respectively.

## Discussion

 This study provides new information about the frequency, severity, and resulting interference of fatigue in patients with SCD. Our results showed that more than 40% of adults with sickle cell disease had severe depression, of whom 67 % had evidence of clinical symptoms. Similar to findings by Ekinci et al. in a review study, the majority of the available studies have shown that patients with SCD have a higher risk of depressive symptoms^5^. In a study conducted by Jerrell et al., 46 % of patients with sickle cell disease were diagnosed with a depressive disorder^6^. While Benton et al. reported that only 12.5% of patients with SCD had major depressive disorder and Graves et al. stated that the majority of participants (83.9%) had low risk of depression^7^^,^^8^.

Although the risk factors of depression in SCD patients are not entirely known, there is evidence indicating that some disease-related factors such as frequency of pain episodes, higher frequency of vaso-occlusive pain attacks, frequency of visits for acute chest syndrome per year may develop disease-related complications and may have an influence on depressive syndrome. Even family status can be associated with psychological distress among these patients ^5^^- ^^6^^, ^^9^^. ^

Fifty-five percent of the participants in this study were found to have severe anxiety. So, based on the available evidence, 91.8% of SCD patients were symptomatic. Although most studies have confirmed the presence of anxiety in SCD patients, there are some differences in their reported levels *of anxiety* ^5^^,^^7^^- ^^10^. For example, Graves et al. reported the majority of respondents had scores below the clinical thresholds, indicating low risk for anxiety^8^. Levenson et al. also demonstrated that anxiety in SCD occurs at rates comparable to or slightly greater than healthy populations^20^. Previous studies explained that living with a chronic stigmatizing disease, the unpredictable painful nature of SCD, frequent hospitalizations, fear of worsening of the condition and worries about the future social life as an individual associated with SCD appear to contribute to an increased risk of anxiety disorders ^20^^- ^^22^. 

Brown & Thorsteinsson study indicated that acute and chronic stressors are main causes of fatigue among healthy adults^23^, and in our research more than half of the SCD patients (46.4%) showed severe stress and a very tiny percentage of whom was asymptomatic. Mahdi et al. and Ahmadi et al. also stated that 49% and 52% of the SCD patients had high level of stress^10^^-^^11^. The above observed differences may be due to possible differences in sampling procedures, sample size, inclusion criteria, sample characteristics and applied screening tools. 

As stated in previous publications, most (64.9%) of the SCD patients showed feeling of tiredness in our study^3^^-^^4^^,^^12^^,^^24^. To the best of our knowledge, most of the studies confirm that fatigue exist among patients with SCD.

 Based on our findings, the behavioral variables (depression, anxiety and stress) were significantly correlated with fatigue. The results of the two previous studies are consisted with our findings ^3^^, ^^4^^.^ Moreover, the comparison of patients with symptoms of stress and depression with those without symptoms mentioned above indicated that the two groups have significant differences, except for anxiety. In other words, patients with signs of fatigue had significantly more depressive symptoms and stress compared to those without these symptoms. But, there were no statistically significant differences between the groups with and without signs of anxiety. Although these results have not been obtained by other studies, they seem reasonable enough. 

The other results of this study demonstrated the relationships between fatigue and depression, stress and blood transfusion. In addition, fatigue and stress had significant positive correlation with renal diseases. Meanwhile, patients suffering from heart disease reported further levels of anxiety and stress. The relationship between stress and splenic diseases was also observed in this study. The obtained results are in agreement with the results taken from Simon et al study in which they found that when the severity of a disease is mild, the incidence is low for symptoms such as depression, anxiety, etc^25^.

 Results of stepwise multiple regression analysis showed that first model depression has played the most important role in predicting fatigue. Like previous studies, one of the most common residual symptoms of depression is fatigue, and patients with fatigue are more likely to get depressed^26^. Moreover, depression is a highly comorbid condition that is not uncommon in chronic illnesses^24^, and is strongly linked to fatigue. In SCD, depression tends to occur at higher rates (18%–27)^20^, but the nature of the relationship between depression and fatigue is unclear, and differentiating fatigue-related depression from physical illness symptoms can be challenging^3^. Therefore, it can be said that depression was a significant predictor for fatigue.

In next model, the blood transfusion variable increased the fatigue predictive power for about 10%. Blood transfusions are often used as a potential treatment for fatigue in anemic palliative care patients. However, the use of validated outcome measures has been found to be a common problem^27^. Patients with SCD are equally at risk for hypoxemia if their hematocrit is too high, resulting in hyperviscosity^3^. It can result from simple and partial exchange transfusions that are commonly used for prevention and treatment of certain complications of SCD such as stroke and priapism^28^. Specified hypoxemia has also been associated with fatigue^29^. 

Finally, the third and fourth variables, kidney disease and work status, resulted in 36% changes in fatigue status of SCD patients. There are a number of factors that may perpetuate clinically significant fatigue among individuals with chronic kidney disease, including sleep disorders, depression, sedentary lifestyle, anemia, and chronic inflammation^30^. While and Mullen in their qualitative study on SCD patients stated that the majority of participants complained of being tired, not having energy, wanting to sleep, and often being unable to carry out activities of daily living. Fatigue often interfered with the ability to carry out daily activities^31^. Nevertheless, reporting more fatigue by working and student patients is not unexpected. 

Several limitations need to be mentioned when interpreting the study. First, the sample size was small, which limits the generalizability of the findings. Second the cross-sectional design limited the ability to examine fatigue over time. Third results are from one institution which limits representativeness to other patients with sickle cell disease in other settings. A healthy group for comparison of fatigue levels was not included.

## CONCLUSION

 Overall, more than 50% of study participants with SCD experienced depression, anxiety, stress and fatigue. There was a positive correlation between depression, anxiety, stress and fatigue in this study. The survey also revealed that fatigue was more common among those experienced more depression and stress. The results of the study indicated that SCD patients who had depression, underwent blood transfusions, had SCD-related kidney complications, students and working people experienced more fatigue. 

Fatigue has been detected as a symptom of anxiety, depression, stress, and also a symptom of the disease itself. So, if fatigue is present, it is important to recognize the existence of these conditions or vice versa.

Routine assessment and improved management of fatigue, effective interventions to reduce fatigue, are highly recommended for patients with SCD. Further studies to identify the trajectory of fatigue and influencing factors are required.
